# Estimating Long-Term Survival of Critically Ill Patients: The PREDICT Model

**DOI:** 10.1371/journal.pone.0003226

**Published:** 2008-09-17

**Authors:** Kwok M. Ho, Matthew Knuiman, Judith Finn, Steven A. Webb

**Affiliations:** 1 School of Population Health, University of Western Australia and Royal Perth Hospital, Perth, Western Australia, Australia; 2 School of Medicine, University of Western Australia and Royal Perth Hospital, Perth, Western Australia, Australia; 3 School of Population Health, University of Western Australia, Crawley, Western Australia, Australia; Oregon Health & Science University, United States of America

## Abstract

**Background:**

Long-term survival outcome of critically ill patients is important in assessing effectiveness of new treatments and making treatment decisions. We developed a prognostic model for estimation of long-term survival of critically ill patients.

**Methodology and Principal Findings:**

This was a retrospective linked data cohort study involving 11,930 critically ill patients who survived more than 5 days in a university teaching hospital in Western Australia. Older age, male gender, co-morbidities, severe acute illness as measured by Acute Physiology and Chronic Health Evaluation II predicted mortality, and more days of vasopressor or inotropic support, mechanical ventilation, and hemofiltration within the first 5 days of intensive care unit admission were associated with a worse long-term survival up to 15 years after the onset of critical illness. Among these seven pre-selected predictors, age (explained 50% of the variability of the model, hazard ratio [HR] between 80 and 60 years old = 1.95) and co-morbidity (explained 27% of the variability, HR between Charlson co-morbidity index 5 and 0 = 2.15) were the most important determinants. A nomogram based on the pre-selected predictors is provided to allow estimation of the median survival time and also the 1-year, 3-year, 5-year, 10-year, and 15-year survival probabilities for a patient. The discrimination (adjusted c-index = 0.757, 95% confidence interval 0.745–0.769) and calibration of this prognostic model were acceptable.

**Significance:**

Age, gender, co-morbidities, severity of acute illness, and the intensity and duration of intensive care therapy can be used to estimate long-term survival of critically ill patients. Age and co-morbidity are the most important determinants of long-term prognosis of critically ill patients.

## Introduction

Demand for intensive care unit (ICU) services is increasing [1], and at a rate that is higher than the average for all health care services [2]. Increase in treatment and monitoring technology, patients' expectations, and ageing population all contribute to this increased demand for intensive care services [1]. Indeed, intensive care is increasingly being provided to older and sicker patients, whom in the past were not treated in intensive care [3]. Intensive care services accounted for 10% of the US$2.1 trillion total health expenditures on health care in the United States in 2006 [4] and has been estimated to cost more than £700 million in the United Kingdom in 1999 [5]. The cost of intensive care services coupled with increasing demand provides the rationale for improved modelling of outcomes of critically ill patients.

Long-term survival after critical illness is increasingly being recognized as an important outcome in assessing effectiveness of new therapy [6]. In order to control for confounding and bias in assessing long-term survival of critically ill patients in a clinical trial, a risk adjustment tool that can objectively estimate long-term survival is essential. From a clinical perspective, many patients and clinicians are also interested in knowing the long-term survival outcome after critical illness, in addition to other information such as burden of treatment and quality of life after recovery, when making treatment decisions in the ICU. Although many clinicians may foretell patient hospital survival outcome more accurately than some objective prognostic models [7], treatment decisions made by clinicians do vary considerably with their practice style and work experience [8]–[10]. The strategy of continuing intensive treatment for all patients until death will reduce the need for patients and clinicians to make difficult treatment decisions and may improve the survival time of some. This strategy is, however, expensive and undesirable by imposing excessive burden of treatment on those who have a very poor prognosis [11]. For example, initiating acute renal replacement therapy in critically ill patients with less than 10% probability of 6-month survival was estimated to cost US$274,000 (£137,000) per quality-adjusted life year saved [12].

The SUPPORT investigators from the United States and Wright et al. from the United Kingdom published two prognostic models that were based on age, severity of acute illness and admission diagnosis to estimate 6-month and 5-year survival of critically ill patients, respectively [13], [14]. The utility of latter model is, however, limited by its ability to classify 5-year survival probabilities only into three risk categories when the calculated risk score is either <70, 70–80, or >80 [14]. This model also did not consider the potential effect of detailed co-morbidity data on long-term survival of critically ill patients beyond the usual assessment included in the Acute Physiology and Chronic Health Evaluation (APACHE) score [14], [15]. There is currently no prognostic model that is available to estimate the survival of critically ill patients beyond 5 years after the onset of critical illness. Furthermore, the relative importance of age, co-morbidity, and severity of acute illness in determining long-term prognosis of critically ill patients also remains unknown. In this study we examined the long-term survival of 11,930 critically ill adult patients who survived at least 5 days and developed a new prognostic model (**P**redicted **R**isk, **E**xisting **D**iseases, and **I**ntensive **C**are **T**herapy: the **PREDICT** model) to estimate their median survival time and long-term survival probabilities.

## Methods

### The characteristics of the cohort

This cohort study utilized the clinical database of the ICU at Royal Perth Hospital (RPH) in Western Australia. RPH is the largest tertiary university teaching hospital in Western Australia and the 22-bed ICU admits patients of all specialties except liver transplantation and captures over 40% of all critically ill patients in Western Australia. The database analyzed in this study includes details of all ICU admissions between 1989 and 2002, including demographic factors, admission diagnosis, admission source, severity of acute illness as measured by APACHE II scores based on the worst first 24-hour ICU data [15], daily organ failure assessment and supportive therapy required [16], and ICU and hospital survival outcome.

In this study the patients with a diagnosis excluded from the original APACHE II cohort (e.g. coronary artery graft surgery, burns, snake bite)[15], those who resided outside Western Australia at the time of ICU admission (who could not be followed for survival), readmissions after the first ICU readmission, patients who were younger than 16 years old, and patients who did not survive more than 5 days during their hospitalization of the index ICU admission were excluded. The data were reviewed for internal consistency annually, and there were no patients with missing hospital mortality data. Some of the other details of this cohort have been described in our previous publications [16]–[18].

The ICU clinical database was linked to the Western Australian hospital morbidity and mortality databases using probabilistic matching [16], providing information on patients' co-morbidities as recorded in all private and public hospital admissions including any prior ICU admissions up to 5 years before the index ICU admission. A relatively long five-year ‘look back’ period was chosen in this study to capture all existing co-morbidities of each patient. We ascertained the presence of co-morbidities in the Charlson co-morbidity index (**Table 1**) using the published ICD-9-CM and ICD-10-AM coding algorithms [16], [19]. We did not assign a co-morbidity to a patient if that condition was diagnosed during the hospitalization of the index ICU admission. The proportions of invalid (false positive) and missed links (false negatives) between Western Australian hospital morbidity and mortality databases were evaluated several years ago, and both false positives and negatives were estimated to be 0.11% [20].

**Table 1:** Charlson co-morbidity index component and its weighting.

Co-morbidity	Weighting
Myocardial infarction	1
Congestive heart failure	1
Peripheral vascular disease	1
Cerebrovascular disease	1
Dementia	1
Chronic pulmonary disease	1
Connective tissue disease	1
Peptic ulcer disease	1
Mild liver disease	1
Diabetes mellitus	1
Hemiplegia	2
Moderate or severe renal disease	2
Diabetes with end-organ damage	2
Any tumour	2
Leukemia	2
Lymphoma	2
Moderate to severe liver disease	3
Metastatic solid tumour	6
AIDS	6

The survival status of the patients was assessed on 31 December 2003 and the length of follow up ranged from 1 year to 15 years with an average of 6 years. Western Australia is geographically isolated and has a very low rate of emigration (<0.03% in 2002)[16], and as such, lost to long-term survival follow-up by the Western Australian mortality database is likely to be very low. The data analyzed had the patient name and address removed and the study was approved by the RPH Ethics Committee and the Western Australian Confidentiality of Health Information Committee (CHIC).

### Development of the model

The prognostic model was fitted using Cox proportional hazards regression [21], restricting predictors to factors that were likely to be important predictors of long-term survival of hospitalized patients [13], [14], [22], [23]. These pre-selected factors included age [14], [22], gender [22], APACHE II predicted mortality risk [13]–[15], Charlson co-morbidity index [19], [23], days of mechanical ventilation, acute renal replacement therapy or hemofiltration, and vasopressor or inotropic therapy during the first 5 days of the index ICU admission [13]. The APACHE II predicted mortality was chosen as a measure of severity of acute illness because it is widely used and summarizes the diagnosis, acute physiologic derangement within the first 24 hours of ICU admission, severe co-morbidities, and whether the ICU admission is after elective or emergency surgery. Our previous study also showed that the APACHE II predicted mortality has a very stable performance in this cohort over the past 10–15 years [17]. Although age and severe co-morbidities are already used to calculate the APACHE II predicted mortality [15], these two factors may still have a profound effect on long-term survival over and beyond the weightings used in the APACHE II predicted mortality [14], [22], [23]. As such, both age and Charlson co-morbidity index were used as separate predictors in additional to the APACHE II predicted mortality in this prognostic model. These seven predictors were also chosen because they are often recorded in the administrative databases of many ICUs, and as such, it is possible for other ICUs to validate this model using their data [24].

**Figure 1:**
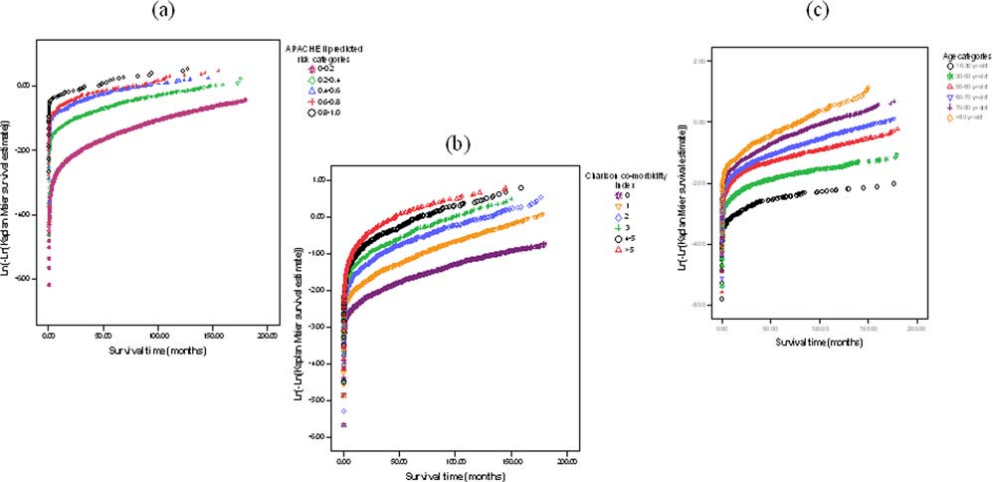
The proportional hazards assumption of the predictors in the Cox model was assessed by plotting the logarithm of the negative logarithm of the Kaplan Meier survivor estimates and the assumption was found to be acceptable for the three pre-selected continuous predictors; APACHE II predicted mortality, Charlson co-morbidity index, and age. (a) Graph assessing the proportionality of hazards associated with severity of acute illness measured by the APACHE II predicted mortality risk categories (0–20%, 20–40%, 40–60%, 60–80%, 80–100%). (b) Graph assessing the proportionality of hazards associated with co-morbidities measured by Charlson co-morbidity index categories (0, 1, 2, 3, 4–5, >5). (c) Graph assessing the proportionality of hazards associated with age measured by age categories (16–30, 30–50, 50–60, 60–70, 70–80, >80 years old)

The proportional hazards assumption of the continuous predictors in the Cox model was assessed and found to be acceptable (**Figure 1a, 1b, 1c**). During the modelling process, we avoided categorizing continuous predictors [24], [25] and allowed a non-linear relationship with hazard of death using a 6-knot restricted cubic spline function [25]. The relative contribution of each predictor was assessed using the chi-square statistic minus the degrees of freedom [25]. The discrimination performance of the model was assessed with the c-index, which is a generalization of the c-statistic or the area under the receiver-operating characteristic curve, allowing for censored data [25], [26]. In this study, a c-index between 0.70 and 0.80 was regarded as acceptable and a c-index above 0.80 was regarded as excellent [27]. Using the Design library in S-PLUS software (version 8.0, 2007. Insightful Corp., Seattle, Washington, USA), the c-index was computed and adjusted for optimism (arising from using the same data to develop the model and assess its performance) by a bootstrap technique to penalise for possible over-fitting, with 200 re-samples and at least 200 patients per risk group [25], [28]. The bootstrapping technique was used in this study instead of splitting the data into development and validation data set because this method is regarded as most data ‘efficient’ and accurate in developing a prognostic model [25]. Model calibration (similarity of predicted risks and proportions actually dying) was assessed graphically and used a bootstrap re-sampling to construct a bias-corrected calibration curve and its slope [25], [29]. Nagelkerke's R^2^ (a generalized measure of the percentage of the variance in survival accounted for by the model) was computed to assess the overall performance of the model [25], [30]. The performance of the model was assessed over the full 15 years of follow-up, when follow-up was restricted to a maximum of 5 years for each patient, and also when only patients admitted after 1997 were considered.

A nomogram was developed for the model that generates the median survival time and selected annual survival probabilities by adding up the scores for each of the seven predictors [25]. The use of the nomogram and how each predictor may affect a patient's long-term prognosis is described for a selection of typical patient scenarios. In particular, these scenarios were selected to illustrate how the long-term prognosis of a patient can be different from the short-term prognosis. Nevertheless, the results of the nomogram should only be considered as an average estimate of patients with similar characteristics and not be used for individual patients.

## Results

### Characteristics of the cohort

The study cohort consisted of a heterogeneous group of critically ill patients, with elective surgery including heart valve surgery, urology, gastrointestinal and spinal surgery accounting for 36.2% of all ICU admissions. The emergency admissions consisted of patients with multiple trauma (8.5%), isolated head trauma (2.5%), acute myocardial infarction, congestive heart failure, cardiac arrhythmias, or cardiogenic shock (7.4%), hypovolemic or hemorrhagic shock (0.8%), drug overdoses (7.2%), subarachnoid or intracranial hemorrhage (5.1%), sepsis (4.3%), pneumonia or aspiration (3.7%), obstructive airway diseases (2.1%), cardiorespiratory arrest (4.0%), gastrointestinal hemorrhage, perforation or obstruction (2.4%), and other medical and surgical emergencies. Details of this cohort including demographic factors, co-morbidities, severity of acute illness, and the length of ICU and hospital stay are described in **Table 2**.

**Table 2:** Characteristics of the cohort (n = 11,930).

Variables	Mean (median, standard deviation), unless stated otherwise
Age, yrs	53.8 (57.0, 19.0)
Gender (male/female), no. (%)	7489 (62.8)/4441 (37.2)
Elective surgery admission, no. (%)	4318 (36.2)
APACHE II score	13.7 (13.0, 6.8)
APACHE II predicted mortality, %	14.5 (7.0, 17.8)
No. of APACHE co-morbidities	0.1 (0, 0.3)
(a) Cardiovascular, no. (%)	592 (5.0)
(b) Respiratory, no. (%)	210 (1.8)
(c) Renal, no. (%)	109 (0.9)
(d) Immunosuppressed, no. (%)	197 (1.7)
(e) Liver, no. (%)	76 (0.6)
No. of Charlson co-morbidities	0.8 (0, 1.2)
Charlson co-morbidity index	1.0 (0, 1.7)
Length of ICU stay, days	5.6 (3.0, 8.3)
Length of hospital stay, days	20.3 (13.0, 25.9)
No. of patients mechanically ventilated (%) #	8034 (67.3)
No. of patients on inotrope (%) #	3921 (32.9)
No. of patients on dialysis (%) #	608 (5.1)
No. of ICU survivor (%)*	11557 (96.9)
No. of hospital survivor (%)*	11101 (93.1)
No. of survivor/total no. of patients followed up (%)	
(a) at 1-year	10334/11101 (93.1)
(b) at 3-year	8031/10019 (80.2)
(c) at 5-year	6109/8212 (74.4)
(d) at 10-year	2609/4238 (61.6)
(e) at 15-year	441/887 (49.7)

# During the first 5 days in ICU.

* Excluding patients died within 5 days of ICU admission.

ICU, intensive care unit.

APACHE, Acute Physiology and Chronic Health Evaluation.

### Effect of the Predictors on Hazard of Death

**Figure 2:**
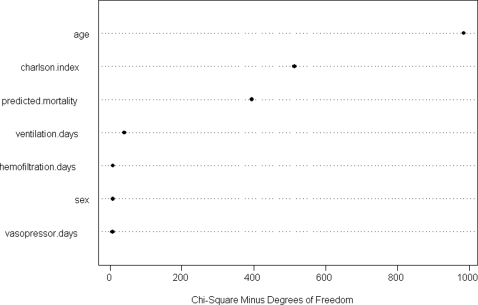
Contribution of each predictor in predicting the survival time in the Cox proportional hazards model.

**Figure 3:**
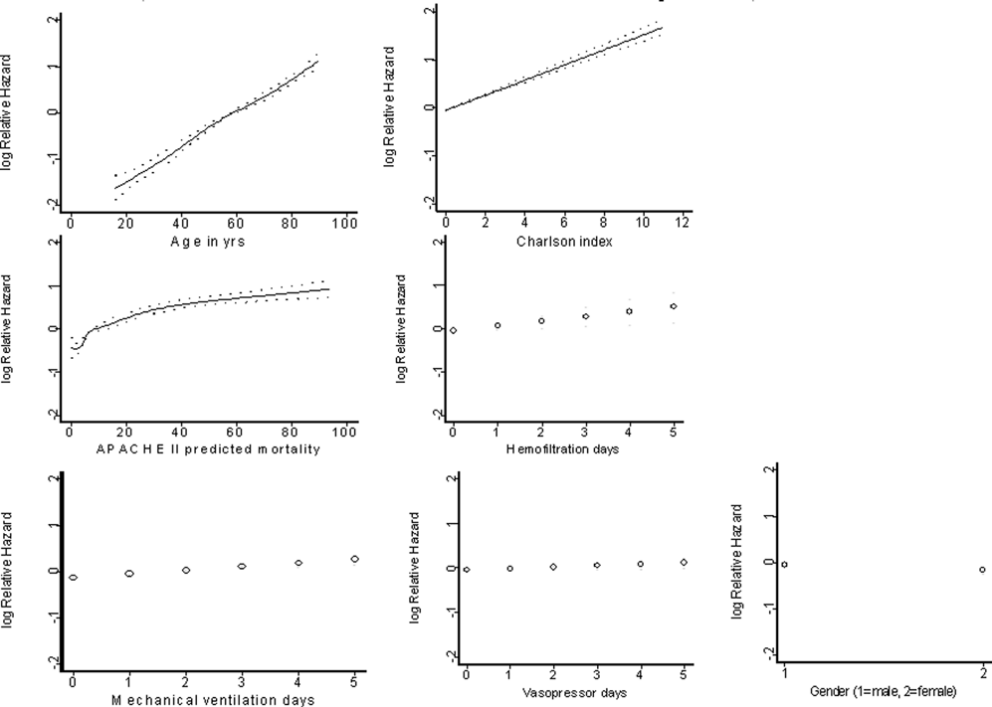
The relationship between relative hazard and each predictor after adjusting for other predictors in the model.

**Figure 4:**
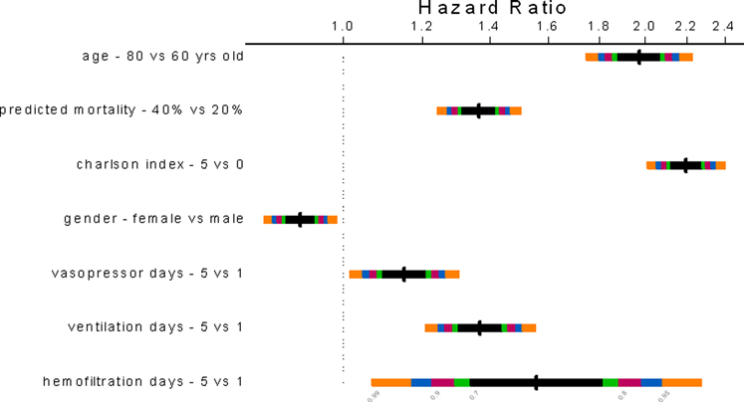
The estimated (adjusted) hazard ratios and multilevel confidence bars (0.70 as illustrated by the black bar to 0.99 as illustrated by the orange bar) for the effects of predictors in the model are summarized in the figure below. An increase of 20 years of age and an increase in Charlson co-morbidity index from 0 to 5 approximately doubled the risk of death. Doubling the APACHE II predicted mortality from 20% to 40% increased the relative risk of death by about 30 to 40%. Similarly, increased the number of days of intensive care therapy from 1 to 5 increased the relative risk of death by between 10% and 50%.

Among all the seven pre-selected predictors in the model, age (50%), co-morbidity as measured by Charlson co-morbidity index (27%), and severity of acute illness as measured by the APACHE II predicted mortality (20%) made the strongest contributions in predicting survival time (**Figure 2**). After adjusting for other predictors, the log hazard of death increased linearly with age, Charlson co-morbidity index, and the number of days of vasopressor or inotropic therapy, mechanical ventilation, or hemofiltration therapy (**Figure 3**). The relationship between the APACHE II predicted mortality and log hazard of death was non-linear with a steep effect when the APACHE II predicted mortality was less than 10% and a smaller effect when it was more than 10%. The estimated (adjusted) hazard ratios for the seven predictors are summarized in **Figure 4**.

### Clinical Application of the Model

**Figure 5:**
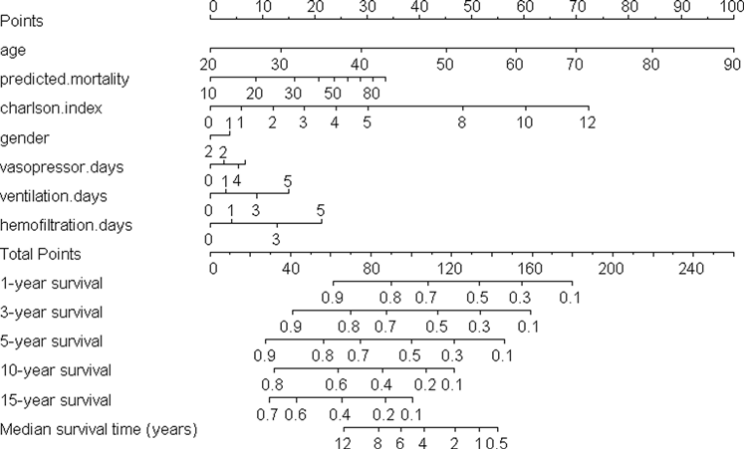
Nomogram for predicting long-term survival probabilities and median survival time. Note: gender: 2 = female, 1 = male. Predicted.mortality = APACHE II predicted mortality in %.

**Figure 5** presents the model in the form of a nomogram that provides the median survival time and long-term survival probabilities corresponding to a particular total score. The total score for a patient is obtained by adding up the scores for each of the seven predictors. We use the following hypothetical but typical patients to illustrate how the nomogram is used and how the short-term prognosis of a patient can be quite different from the long-term prognosis.

**Patient A:**

A 40-year old male, without pre-existing co-morbidities (ie Charlson co-morbidity index = 0), was admitted to the ICU because of septic shock with an APACHE II predicted mortality of 80%. He required vasopressor or inotropic therapy, mechanical ventilation, and hemofiltration therapy during the first 5 days in the ICU.

The gender of this patient scores 5 points, age scores 28 points, Charlson co-morbidity scores zero points, the APACHE II predicted mortality or risk scores 30 points, 5 days of vasopressor or inotropic therapy scores 7 points, 5 days of mechanically ventilation scores 15 points, and 5 days of hemofiltration scores 20 points. The total score of this patient is therefore 105 which gives an estimated median survival time of about 4 years, >70% 1-year survival probability, >50% 3-year survival probability, >45% 5-year survival probability, and >20% 10-year survival probability.

**Patient B:**

A 70-year old female, with chronic obstructive airway disease and non-insulin dependent diabetes mellitus with no end-organ damage (ie Charlson co-morbidity index = 2), was admitted to the ICU because of severe community acquired pneumonia with an APACHE II predicted mortality of 30%. She required vasopressor or inotropic therapy and mechanical ventilation but not hemofiltration during the first 5 days in the ICU.

The gender of this patient scores zero points, age scores 70 points, Charlson co-morbidity index scores 12 points, the APACHE II predicted mortality scores 16 points, 5 days of mechanical ventilation scores 15 points, and 5 days of vasopressor or inotropic therapy scores 7 points. The total score of this patient is therefore 120 which gives an estimated median survival time of about 2 years, 60% 1-year survival probability, 40% 3-year survival probability, 30% 5-year survival probability, and 10% 10-year survival probability.

**Patient C:**

A 80-year old male, with a history of myocardial infarction, congestive heart failure, peripheral vascular disease, cerebrovascular disease, and dementia (ie Charlson co-morbidity index = 5), was admitted to an ICU with bowel perforation and peritonitis with an APACHE II predicted mortality of 30%. He required vasopressor or inotropic therapy and mechanical ventilation but not hemofiltration during the first 5 days in the ICU.

The gender of this patient scores 5 points, age scores 85 points, Charlson co-morbidity index scores 30 points, the APACHE II predicted mortality scores 16 points, 5 days of mechanical ventilation scores 15 points, and 5 days of vasopressor or inotropic therapy scores 7 points. The total score of this patient is therefore 158 which gives an estimated median survival time of <0.5 years, 25% 1-year survival probability, and 10% 3-year survival probability.

### Discrimination and Calibration of the Prognostic Model

**Figure 6:**
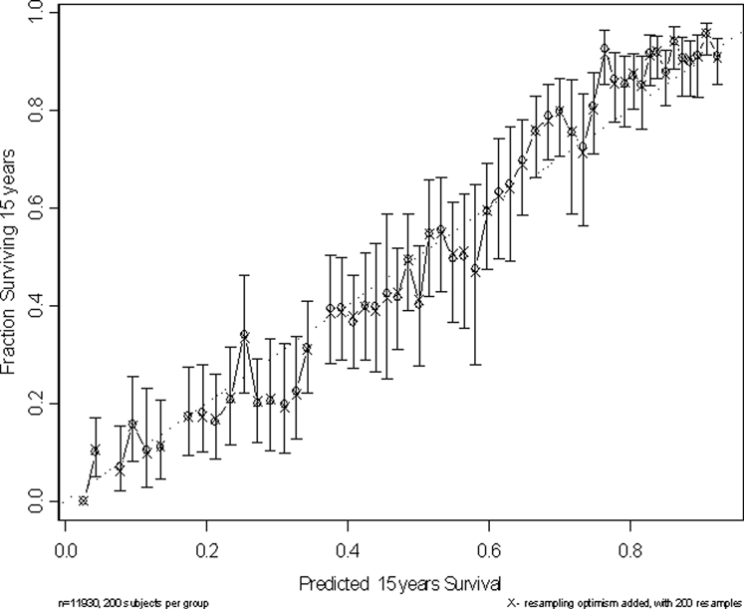
Bootstrap estimate of calibration accuracy for 15-year estimates from the Cox proportional hazards model. Dots correspond to apparent predictive accuracy and x marks the bootstrap-corrected estimates.

The adjusted c-index for this prognostic model was 0.757 (95% confidence interval 0.745–0.769), Nagelkerke's R^2^ was 0.255 and the bias-corrected calibration of the model over a 15-year period was reasonable (slope of the calibration = 0.98)(**Figure 6**). The Nagelkerke's R^2^ remained unchanged and the adjusted c-index only increased marginally when the analysis was restricted to a maximum of 5 years follow up (c-index = 0.759, slope = 0.97) or data after 1997 (c-index = 0.762, slope of the calibration = 0.97).

## Discussion

This study showed that age, gender, co-morbidities (Charlson co-morbidity index), severity of acute illness (the APACHE II predicted mortality), and duration of intensive care therapy or organ support within the first 5 days of ICU admission are important prognostic factors for long-term survival of critically ill patients. To the best of our knowledge, this new prognostic model (**P**redicted **R**isk, **E**xisting **D**iseases, and **I**ntensive **C**are **T**herapy: the **PREDICT** model) is the first preliminary prognostic model that can be used to estimate the median survival time and long-term survival probabilities of critically ill patients up to 15 years after the onset of critical illness.

The current prognostic model has confirmed that age, gender, co-morbidities, severity of acute illness, and duration of intensive care therapy or organ failure are important predictors of 6 months to 5 years survival of hospitalized or critically ill patients [13], [14], [19], [22], [23]. The current model is indeed built on the results of these previous studies but further extended the significance of these risk factors in predicting survival of critically ill patients beyond 6 months to 5 years. This current model also demonstrated that most of these predictors have a relatively linear relationship to the long-term survival probability. More importantly, our results also showed that age and co-morbidities are the most important determinants of long-term prognosis of critically ill patients. This latter finding has at least two significant clinical implications. First, the factors that determine long-term survival of a critically ill patient are different from those that affect short-term prognosis. Previous evidence suggested that diagnosis and acute physiological derangement of a patient are most important in determining hospital survival [15], [31]. In our three hypothetical patients, Patient A has in fact the most severe form of acute critical illness and worst short-term prognosis. Nevertheless, because this patient is younger and has no co-morbidities, this patient has a very reasonable and better long-term prognosis than Patient B and C. If we use the prognostic model developed by Wright et al. [14] to estimate the long-term survival of our three hypothetical patients, Patient B will have the best 5-year prognosis (risk score is estimated to be 68) followed by Patient C (risk score 75) and then Patient A (risk score 87). The lack of detailed co-morbidity data and a heavy emphasis on severity of acute illness in the model developed by Wright et al. is the most likely explanation why our results are different from theirs.

Many clinicians may intuitively consider the intensity of organ failure as very important in affecting a patient's prognosis [32], [33]. Our findings suggest that the effect of acute organ failure on long-term survival is not strong and mostly captured by age, co-morbidities, and the APACHE II predicted mortality on admission to ICU. Our previous studies have also showed that the intensity of organ failure alone is not as important as the APACHE II score in predicting hospital mortality [34], [35]. Therefore, our findings suggest that clinicians should be very careful not to place undue emphasis on the severity of acute illness and intensity of organ failure when making long-term prognostications of critically ill patients.

Second, because the contributions by intensive care therapy are relatively small when compared to age, Charlson co-morbidity index, and the APACHE II predicted mortality, using the data after the first 24 to 48 hours of ICU stay is unlikely to underestimate the final total prediction score significantly (<20 points)(**Figure 5**). Therefore, early estimation of a slightly ‘optimistic’ long-term survival probability and median survival time is feasible after the first 24 to 48 hours of ICU stay; and in patients with either extremes of prognosis, this early estimation is unlikely to be significantly different from the final prediction by collecting all data after five days of intensive care therapy. Nevertheless, the current prognostic model utilizes the APACHE II predicted mortality after ICU admission as a predictor to estimate long-term survival, as such, the model cannot be used, in its current form, as a tool to triage ICU admission.

This study has significant limitations. First, patients' wishes and the anticipated quality of life before and after their critical illness are important factors in making treatment decisions [36], [37]. The median survival time and long-term survival probabilities is only one of the many factors that patients and clinicians may consider in making treatment decisions. Furthermore, the c-statistics of this model is only about 0.76 and this leaves considerable uncertainty in its applicability in predicting long-term survival of individual patients. As such, the predicted survival probabilities of this prognostic model should only be considered as an average estimate of patients with similar characteristics and should not be used for individual patients. Second, evidence suggests that combining an objective prognostic model with physicians' intuition may improve the accuracy of outcome prediction [13]. Whether combining this current prognostic model with physicians' intuition will improve its predictive performance further remains uncertain, but this merits further investigation. Third, although we studied a large cohort of critically ill patients, and also the case-mix, severity of illness, and in-hospital survival of this cohort is very similar to many other ICUs in Australia [38], validation of this model by other ICUs that have access to data linkage is essential to assess its generalizability. Finally, although the APACHE II prognostic model is still widely used for risk adjustment purposes in many ICUs [39], [40], it is possible that using newer prognostic models instead of the APACHE II prognostic model may improve our current model [41]. Similarly, the performance of the current model may be improved if we consider more predictors in the model although this will also increase the complexity of the model. In this regard, we hope that the **PREDICT** model developed in this study will be of value to others who aim to develop a new prognostic model to enhance our understanding of long-term survival of critically ill patients.

In summary, Age, gender, co-morbidities, severity of acute illness, and the intensity and duration of intensive care therapy can be used to estimate long-term survival of critically ill patients. Age and co-morbidity are the most important determinants of the long-term prognosis of critically ill patients. The current prognostic model, the **PREDICT** model, provides a framework for prognostications and risk adjustment when long-term survival of critically ill patients is considered.
